# Author’s Reply: Comments on “Vitamin Pharmacogenomics: New Insight into Individual Differences in Diseases and Drug Responses”

**DOI:** 10.1016/j.gpb.2017.09.002

**Published:** 2017-10-07

**Authors:** Mou-Ze Liu, Hai-Yan He, Wei Zhang

**Affiliations:** 1 Department of Clinical Pharmacology, Xiangya Hospital, Central South University, Changsha 410008, China; 2 Institute of Clinical Pharmacology, Central South University, Hunan Key Laboratory of Pharmacogenetics, Changsha 49410078, China; 3 International Medical Department, Xiangya Hospital, Central South University, Changsha 410008, China; 4 National Clinical Research Center for Geriatrics, Xiangya Hospital, Central South University, Changsha 410008, China

Dear Editor,

We thank the author for making meaningful comments on our recent article [1]. The SNP 772G > A (rs602662) in exon 2 of the gene encoding fucosyl transferase (FUT2) has been found to be related with the alterations in plasma vitamin B_12_ levels. GG carriers possessed lower levels of vitamin B_12_. However, we didn’t know the mechanism behind this association.

The author by referring to the related studies, has provided us a feasible explanation for the *FUT2*-based variations in vitamin B_12_ levels. The ancestral (G) allele allows for normal translation of *FUT2*, resulting in ABH secretor phenotype, which is believed to be associated with *Helicobacter pylori*-induced gastritis or associated with decreased gastric intrinsic factor (GIF) secretion, therefore leading to reduced plasma B_12_ levels. It provides sound evidence for us to believe that genetic polymorphisms may exert their effects on vitamin pharmacokinetics pathways, leading to varied plasma vitamin levels and different clinical consequences.

Although there may be refuted responses on the mechanisms, we thank the author for enlightening us to reveal the pharmacogenomics involved in the mechanisms of vitamin variation.

## Competing interests

The authors have declared no competing interests.
